# Building High Rate Capability and Ultrastable Dendrite‐Free Organic Anode for Rechargeable Aqueous Zinc Batteries

**DOI:** 10.1002/advs.202000146

**Published:** 2020-06-25

**Authors:** Nannan Liu, Xian Wu, Yu Zhang, Yanyou Yin, Chengzhi Sun, Yachun Mao, Lishuang Fan, Naiqing Zhang

**Affiliations:** ^1^ School of Chemistry and Chemical Engineering State Key Laboratory of Urban Water Resource and Environment Harbin Institute of Technology Harbin 150001 China; ^2^ Academy of Fundamental and Interdisciplinary Sciences Harbin Institute of Technology Harbin 150001 China

**Keywords:** aqueous zinc batteries, coparticipation, high rate capability, organic anodes

## Abstract

Aqueous zinc‐ion batteries (ZIBs) are an alternative energy storage system for large‐scale grid applications compared with lithium‐ion batteries, when the low cost, safety, and durability are taken into consideration. However, the reliability of the battery systems always suffers from the serious challenge of the large Zn dendrite formation and “dead Zn,” thus bringing out the inferior cycling stability, and even cell shorting. Herein, a dendrite‐free organic anode, perylene‐3,4,9,10‐tetracarboxylic diimide (PTCDI) polymerized on the surface of reduced graphene oxide (PTCDI/rGO) utilized in ZIBs is reported. Moreover, the theoretical calculations prove the reason for the low redox potential. Due to the protons and zinc ions coparticipant phase transfer mechanism and the high charge transfer capability, the PTCDI/rGO electrode provides superior rate capability (121 mA h g^−1^ at 5000 mA g^−1^, retaining the 95% capacity of that compared with 50 mA g^−1^) and a long cycling life span (96% capacity retention after 1500 cycles at 3000 mA g^−1^). In addition, the proton coparticipation energy storage mechanism of active materials is elucidated by various ex‐situ methods.

## Graphical abstract

An organic anode is employed in a highly stable Zn‐ion battery. The low redox potential of perylene‐3,4,9,10‐tetracarboxylic diimide is beneficial for anode design, and the proton coparticipation energy storage mechanism promotes the rate performance and cycle stability. This designing strategy enables high rate performance and good cycle stability of the Zn‐ion battery.

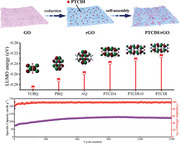

Considering the pressing demand regarding economic and sustainable energy storage system in the context of environmental deterioration and energy dilemma, nonaqueous lithium‐ion batteries have been applied as the most extensive rechargeable energy storage devices on account of their high energy density and long cycle life.^[^ ^1^, ^2^, ^3^ ^]^ However, the restriction on safety and uneven lithium distribution, especially the utilization of noxious organic electrolyte brings about the environmental concern, it is hard to persistently develop large‐scale grid storage applications. Among the various alternative secondary battery candidates, aqueous zinc‐ion batteries (ZIBs) based on Zn metal anode have garnered intensive interests owing to earth abundant resources, cost benefit, and environmental friendliness.^[^ ^4^, ^5^, ^6^ ^]^ To date, tremendous research efforts have been made to seek for potential cathodes, but on the basis of excessive Zn anode,^[^ ^7^, ^8^, ^9^, ^10^ ^]^ resulting in the superficial discharge depth and insufficient utilization of Zn metal, whose results are inauthentic when applied in practical energy storage devices.

In fact, the Zn metal anodes are under continual stripping/plating dynamic processes during charge and discharge, the safety and reliability of the battery systems always suffer from serious challenge of the large Zn dendrite formation caused by uneven Zn electrostripping/electroplating.^[^ ^11^, ^12^ ^]^ Even some dendrites could detach from current collectors before piercing the separators and turn into “dead Zn,” thus losing their capacities. Moreover, the occurrence of the side reactions and water consumption also could induce electrode pulverization and hydrogen evolution, further bringing out the inferior cycling stability as well as safety risks.^[^ ^13^ ^]^ In past few years, there have been some studies associated with depressing Zn dendrites, like electrodepositing Zn on 3D porous Cu skeletons or constructing a porous CaCO_3_ nanolayer to ensure uniform Zn stripping/plating.^[^ ^13^, ^14^ ^]^ Despite that high energy density for full cells has been obtained, the growth of Zn dendrites has not been fundamentally solved.

Integrated with feasibility, excavating alternative nonmetal anodes should be an effective approach to shun substantial issue from Zn metal anodes.^[^ ^15^, ^16^ ^]^ Cheng et al. ever reported the Chevrel phase Mo_6_S_8_ as inorganic anode materials for aqueous zinc‐ion batteries, whereas due to the limitation of its zinc‐intercalating reaction mechanism, low cycle life span and the inferior rate capability (around 60 mAh g^−1^ at a current density of 180 mA g^−1^ after 150 charge–discharge cycles) need to be further boosted.^[^ ^17^ ^]^

Organic molecular materials have drawn attention to divalent cation storage because of intrinsic ductility and soft lattice, which enable molecular reorientation to meet facile ion intercalation, and weak intermolecular van der Waals forces overshadow Coulomb repulsion to mitigate sluggish solid‐state diffusion, generating high specific capacity, structural diversity, and economic friendliness. The features make organic molecules as the sustainable alternative to conventional inorganic electrode materials. However, the property of high discharge voltage makes organic molecules as potential cathode candidates, which lacks flexibility in designing nondendrite anodes, and the alternative organic anodes with low reduction potential should be further explored.

It has been known that the energy of the lowest unoccupied molecular orbital (LUMO) calculated by density functional theory of the compound is linearly related to the first‐stage discharge voltage. Herein, the density functional theory is utilized to calculate the LUMO energy levels of a series of common conjugated carbonyl compounds, revealing the reason that perylene‐3,4,9,10‐tetracarboxylic diimide (PTCDI) is potential as an organic anode in aqueous ZIBs. Furthermore, compared with other organic materials, the PTCDI organic electrode provides the high electron mobility and circumvents the dissolution of discharge products. Under the synergistic effect between the PTCDI and reduced graphene oxide (rGO), the assembled batteries achieve superior rate capability and a long cycling life span, even at discharge current of 5000 mA g^−1^, a high specific capacity of 121 mAh g^−1^ is obtained, retaining 95% of that compared with 50 mA g^−1^, the gap of discharge capacity from 50 to 5000 mA g^−1^ was minuscule. In addition, our research on full battery (commercial prussian blue (PB) as cathode) has demonstrated a suitable reaction potential of 0.95 V when the PTCDI accommodates Zn cations, and could also maintain 75% capacity at 200 mA g^−1^ with the 150 cycles. More importantly, different from general Zn interlayer mechanism occurring on inorganic material,^[^ ^18^, ^19^ ^]^ the PTCDI/rGO electrodes show the protons and zinc ions coparticipant phase transfer mechanism, contributing faster electrochemical reaction rate and reaction kinetics, which could be a preferable choice in terms of rate capability.

To avoid the decrease of electrical conductivity after the polymerization of organic molecules, and thus affect the discharge potential, the PTCDI was in situ synthesized by assembling PTCDI on the surface of rGO with *π*–*π* interaction, accompanying the chemical oxidation polymerization progress. The hybridization of PTCDI and graphene in nanometer size was successfully realized (PTCDI/rGO), the composite diagram was shown in **Figure** **1**. From scanning electron microscope (SEM) and transmission electron microscopy (TEM), and mapping images of PTCDI/rGO (**Figure** **2**a,b and Figure S1 (Supporting Information)), the PTCDI was evenly grown on reduced graphene oxide with thin nanobelt morphology, whose diameter and length were ≈100 nm and 1 µm, respectively. The highly linked structure of the PTCDI nanoflakes and rGO limited the PTCDI from aggregation and restacking, and the highly porous structure caused by rGO maintained vast active sites and ion diffusion paths. The microstructure of polymer was also demonstrated by the X‐ray diffraction (XRD) patterns (Figure 2c). The PTCDI delivered strong diffraction peaks and the peaks’ position could remain unchangeable after compositing with rGO, although the peak intensity was lower, further confirming that the self‐assembly was realized successfully. The Fourier transform infrared spectroscopy (FTIR) exhibited the characteristic peaks of PTCDI/rGO (Figure 2d), which were located at 1360 and 1590 cm^−1^ assigning to the C—N stretch of the polymer and the C=C stretching vibrations of the benzenoid ring of the PTCDI. The additional peaks observed at 1120 and 1686 cm^−1^ were in relation to C—O band and the characteristic band of C=O of the PTCDI, verifying the existence of PTCDI on the rGO.^[^ ^20^, ^21^ ^]^

**Figure 1:**
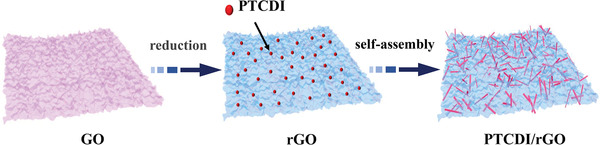
Schematic illustration of preparing the PTCDI/rGO composite.

**Figure 2:**
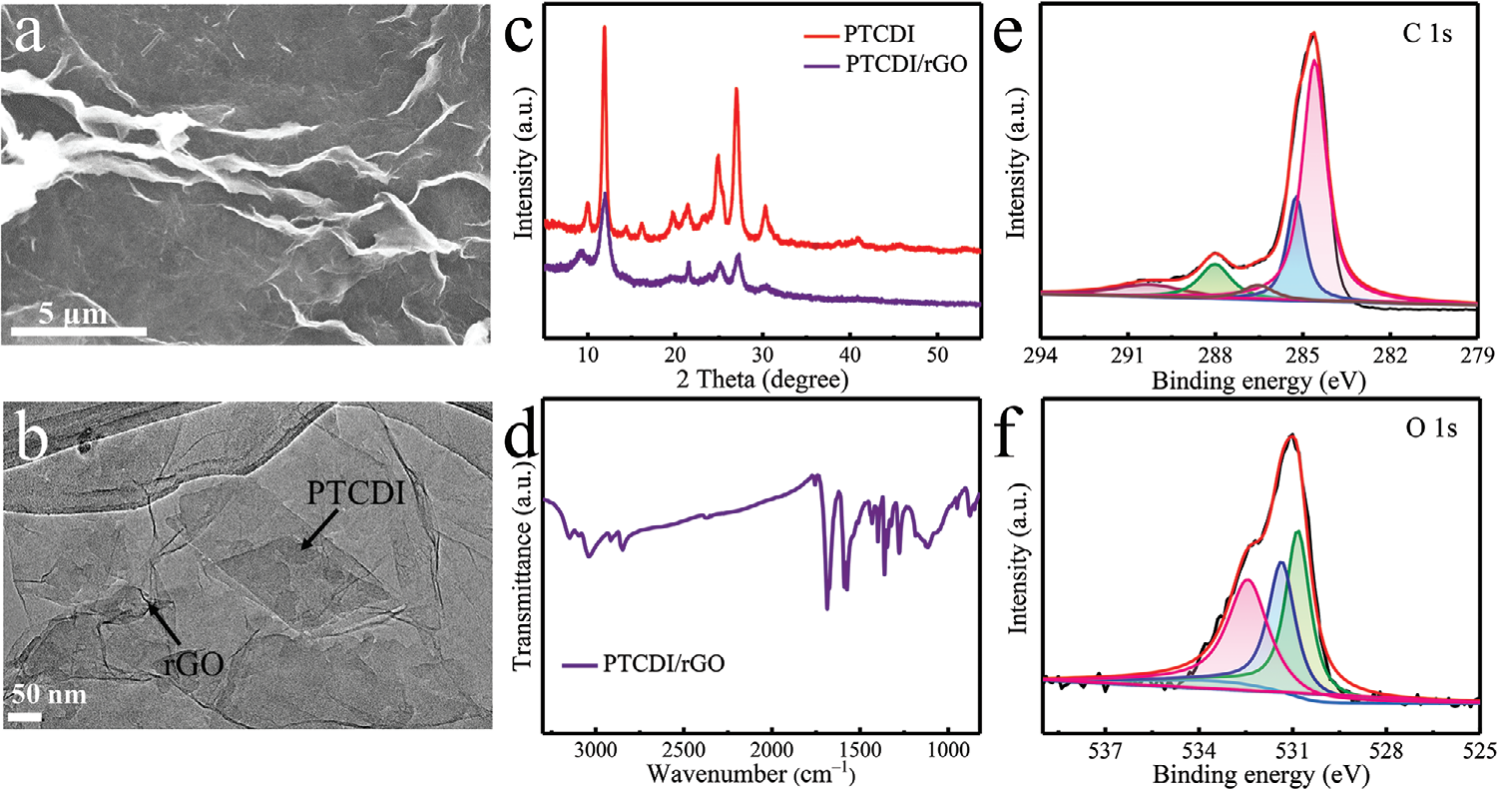
a,b) SEM and TEM images of PTCDI/rGO. c) XRD pattern of PTCDI and PTCDI/rGO composite. d) FTIR comparisons of the PTCDI/rGO composite. XPS spectra of PTCDI/rGO: e) C 1s and f) O 1s.

The X‐ray photoelectron spectroscopy (XPS) spectroscopy was also carried out to characterize the obtained PTCDI/rGO electrode. The overall X‐ray photoelectron spectroscopy (XPS) spectrum showed four peaks that belonged to C, N, O, and F elements (Figure S16a, Supporting Information), while the F was given credit to the polyvinylidene fluoride (PVDF) binder. Figure 2e,f was the high resolution XPS spectra of C 1s and O 1s, respectively. The C 1s spectrum demonstrated four types of C bands, that were C—C (284.4 eV), C—O (285.2 eV), C—N (286.5 eV), and C=O (288 eV) on the polymer chain in PTCDI, and three peaks of O 1s spectrum could be indexed to C=O (diimide of PTCDI), C—O, and —OH, on behalf of surface oxygenic functional groups of rGO. There were two divided peaks in the high‐resolution N 1s XPS spectrum, indicating the existence of C—N and N—H bonds in the side diimide group (Figure S2, Supporting Information).^[^ ^20^, ^22^ ^]^

The electrochemical characterization of PTCDI/rGO composite was studied by fabricating the typical zinc‐ion coin‐type battery, which contained the PTCDI/rGO cathode, Zn anode, and wetted glass fiber separator with aqueous 3 m ZnSO_4_ electrolyte. The splendid rate performance of the assembled batteries was related to the kinetics origin of the electrochemical reactions. Figure S3 (Supporting Information) compared the cyclic voltammetry (CV) curves of different scan rates with the potential range of 0.2–1.8 V. The reduction peaks corresponded to the reaction from PTCDI to Zn–PTCDI, and the oxidation peaks corresponded to the reaction from Zn–PTCDI to PTCDI, respectively. With the scan rate increasing, the characteristic peaks maintained a similar profile, the reduction peaks and the oxidation peaks only slightly shifted to lower or higher potential account for the inconspicuous polarization.

Generally, the relation between peak currents (*i*) and scan rates (*v*) is as below

(1) logi=blogv+loga

where *a* and *b* refer to adjustable parameters. When *b* value is close to 0.5, it indicated that ionic diffusion is the controlling factor of the electrochemical reactions, while the pseudocapacitance occupies critical site for the electrochemical reaction process as if *b* value is near to 1.^[^ ^23^, ^24^ ^]^ By calculating the slopes of Figure S4 (Supporting Information), the results showed that *b* values of total four peaks were ≈1, implying that the battery chemistry behavior of the PTCDI/rGO batteries was dominated by the pseudocapacitance, which was possible to bring about the high rate electrochemical performance of the PTCDI/rGO cathode.

**Figure** **3**a elucidated the rate performance of the PTCDI/rGO electrode at a wide range of current densities from 50 to 5000 mA g^−1^. A high discharge capacity of 121 mAh g^−1^ was obtained at a high current density of 5000 mA g^−1^, retaining 95% of that compared with 50 mA g^−1^, the gap of discharge capacity from 50 to 5000 mA g^−1^ was minuscule. Furthermore, the capacity could reachieve 138 mAh g^−1^ when the current density recovered to 50 mA g^−1^, and the discharge capacity was able to resume to 132 mAh g^−1^ and achieve a 98% capacity retention after 1000 cycles with the following cycles at 1000 mA g^−1^. This characteristic of the excellent rate capability of PTCDI/rGO could meet the pragmatic demand for high energy density and rapid charge/discharge. However, by contrast, the bare PTCDI cathode performed an inferior rate capability, even at a current density of 50 mA g^−1^, and only gained a low discharge capacity of 48 mAh g^−1^. The reason could be attributed to the fact that the existence of graphene promoted uniform dispersion of the PTCDI nanobelts, which decreased the aggregation of the PTCDI and increased the number of available active sites. Meanwhile, the conductive network of rGO satisfied the frequent transport of electrons to PTCDI and met the fast charging and discharging ability of PTCDI/rGO. In addition, the capacity of bare rGO was also tested to eliminate the misunderstanding of higher discharge capacity of PTCDI/rGO. As illuminated in Figure S5 (Supporting Information), rGO contributed only less than 5 mAh g^−1^ based on the mass of PTCDI, stating that the effect of graphene on capacity is negligible.

**Figure 3:**
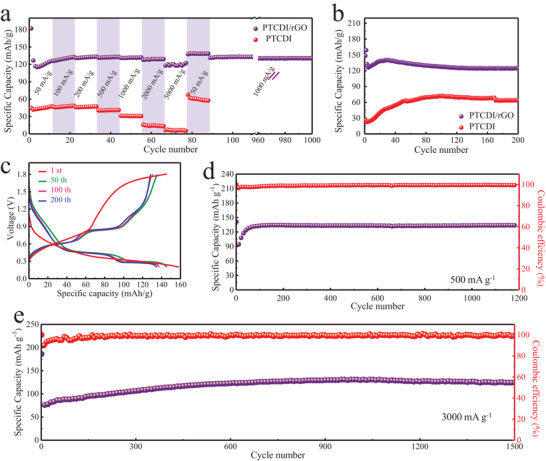
a) The rate capability of PTCDI/rGO. b) Cycling performance of PTCDI and PTCDI/rGO electrodes at 100 mA g^−1^. c) Galvanostatic charge/discharge curves of the PTCDI/rGO at 100 mA g^−1^ with the cycle number of 1st, 50th, 100th, and 200th, respectively. d) Cycling performance of PTCDI/rGO electrodes at 500 mA g^−1^. e) Long‐term cycling performance of PTCDI/rGO at a current density of 3000 mA g^−1^.

Apart from the high rate capability, the PTCDI/rGO cathode also exhibited ideal cycling performance. As shown in Figure 3b, the specific capacity of the PTCDI cathode at 100 mA g^−1^ was merely 73 mAh g^−1^ after activation, in contrast with 52% capacity (141 mAh g^−1^) of the PTCDI/rGO. After 200 cycles, both the electrodes performed a stable cycling performance, indicative of favorable stability of the PTCDI in aqueous electrolyte. While the poor electron transfer ability of PTCDI cathode resulted in continuous capacity increase process within 100 cycles, the PTCDI/rGO cathode promptly reached the stable capacity around 20 cycles. The corresponding galvanostatic charge/discharge profile was depicted in Figure 3c, the discharge and charge profiles had two plateaus, respectively, which were in keeping with the CV curves. The curves had good repeatability after 200 cycles, stating the stable cycle performance of PTCDI/rGO cathode. Besides, the cycle performance of PTCDI/rGO cathode at 500 mA g^−1^ was placed in Figure 3d, an initial specific capacity of 141 mAh g^−1^ was gained, and then stabilized at 134 mAh g^−1^ rapidly, as well as maintained that capacity ultimately (after 1200 cycles) with the high capacity retention ratio of 95%.

We further evaluated the stability of the electrode materials at a higher rate of 3000 mA g^−1^. As depicted in Figure 3e, the increment of the capacity during the initial cycle process was attributed to stepwise electrochemical activation of the PTCDI/rGO, which was in accordance with reported materials.^[^ ^25^ ^]^ The capacity could reach as high as 130 mAh g^−1^ after the activation process, and remained at the high stability after 1500 cycles. The Columbic efficiency was ≈100% due to a high reversibility of Zn ion occupying and decorporation.

Furthermore, the dynamics of the ion solid‐state diffusion of PTCDI/rGO was explored by the galvanostatic intermittent titration technique, and the corresponding ion diffusion coefficients (*D*) at the course of discharge/charge of the electrodes were shown in Figure S7 (Supporting Information). The *D* values of PTCDI/rGO electrode over the whole discharge process were between 10^−7^ and 10^−9^ cm^−2^ s^−1^, and the same as the range of *D* values at charge process, which were much higher than other materials ever reported,^[^ ^26^, ^27^ ^]^ reflecting faster ion diffusion rate of PTCDI/rGO cathode. It should be worthwhile noticing that the *D* values exhibited a decreased tendency with the enhancive depth of the discharge and charge, respectively, the stronger interaction between cations and host materials was deemed to be a probable reason.^[^ ^28^, ^29^ ^]^

The dissolution of the electrode materials during the electrochemical process is able to give rise to the decline of capacity and defaming the cycle life. But, the soluble drawback on the PTCDI/rGO was not observed in aqueous electrolyte, which was visually estimated through unchanged coloration of the electrolyte coupled with clean disassembly battery (Figures S8 and S9, Supporting Information). The UV–vis spectroscopy analysis provided further proof for getting an insight into the stability of electrode compounds in aqueous electrolyte. The electrolyte with the PTCDI/rGO electrodes delivered that there were almost no absorption peaks appearing and the status of the electrolyte remained unshift after cycling, verifying that not only the dissolution of redox species was refrained, but the composition of the electrolyte was steady in charge–discharge progress.

Electrochemical impedance spectroscopy was implemented with a frequency range of 0.1 Hz to 1 MHz (Figure S10, Supporting Information), disclosing distinct resistance variation in different electrochemical cycles. According to the Nyquist plots of the pristine and cycled PTCDI/rGO batteries, the charge transfer resistance became lower after cycles, indicative of its favorable reaction kinetics, which was associated with electrochemical activation process.^[^ ^30^, ^31^ ^]^ After the battery went through several cycles, as expected, the resistance values remain nearly unchanged, this phenomenon could give the credit to stability of electrode materials and the reversibility of redox reaction.

It has been shown from the CV curve above that the first discharge platform of PTCDI appears at 0.38 V. To get a closer look at the distinction reason of the electrode potential from other conventional organic electrodes, the density functional theory was applied to calculate the LUMO of several organic materials (**Figure** **4**), and their detailed structural information was placed in Figure S11 (Supporting Information). It is noted that the redox potential can correlate with LUMO energy levels. Among these compounds, PTCDI possessed the highest LUMO energy level (−0.2247 eV), as a result, corresponding to the lowest discharge potential, which made it a promising anode material.

**Figure 4:**
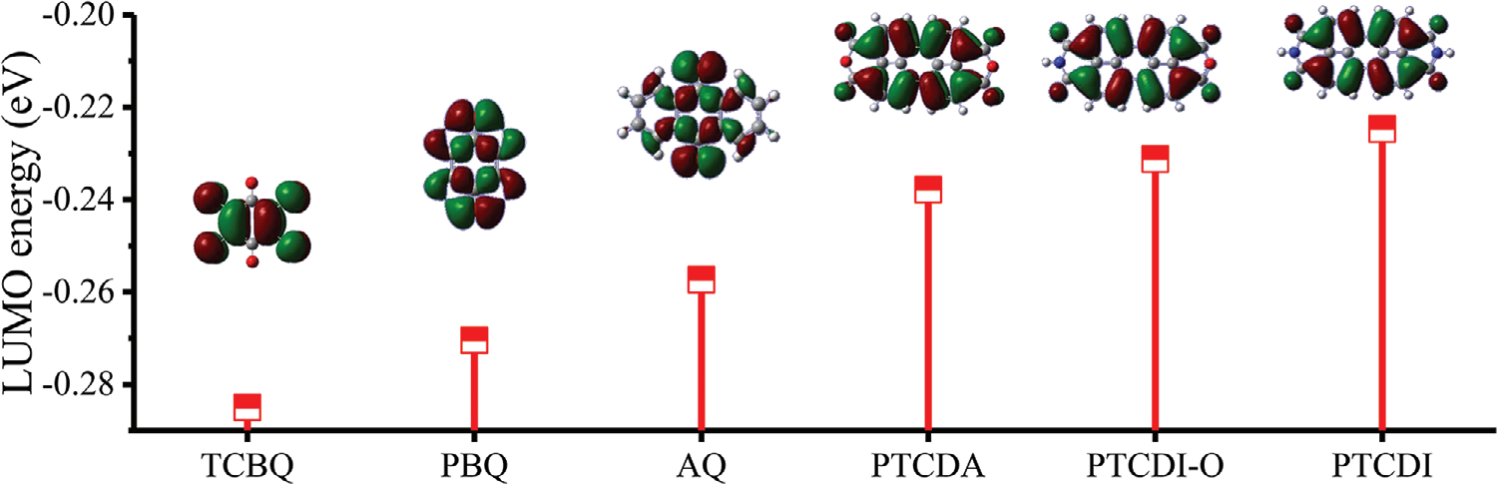
LUMO energy levels calculated by the density functional theory.

The assembled PTCDI/rGO electrodes showed a water participant phase transfer mechanism different from conventional inorganic insertion materials, as depicted in **Figure** **5**a,b. During the initial discharge process to 0.4 V, the carbonyl groups in PTCDI obtained electron to be reduced, in the meantime, the water protons and Zn ions were jointly transferred to the PTCDI at the solid–liquid phase interface of the anode and electrolyte, called as Zn(H)–PTCDI. In the latter discharge process to 0.2 V, the Zn(H)–PTCDI further underwent a metathesis of Zn^2+^ and H^+^ with more cracked C=O bond, where the protons transferred from water were released back into the electrolyte, finally gaining Zn–PTCDI. The reversibility of this mechanism was followed in the later charge progress. The protons reacted with the Zn–PTCDI to form the Zn(H)–PTCDI afresh, which could interact with the PTCDI at original H^+^ occupation sites to remove Zn^2+^ at the charge state of 1.6 V, and the partly cracked carbonyl groups were recovered as the Zn^2+^ were directly released. At the subsequent fully charged stage, the Zn(H)–PTCDI further lost electron, and then more H^+^ interacting with PTCDI got rid, while more concomitant C=O bonds were reserved. The reaction process was described in Figure S12 (Supporting Information).

**Figure 5:**
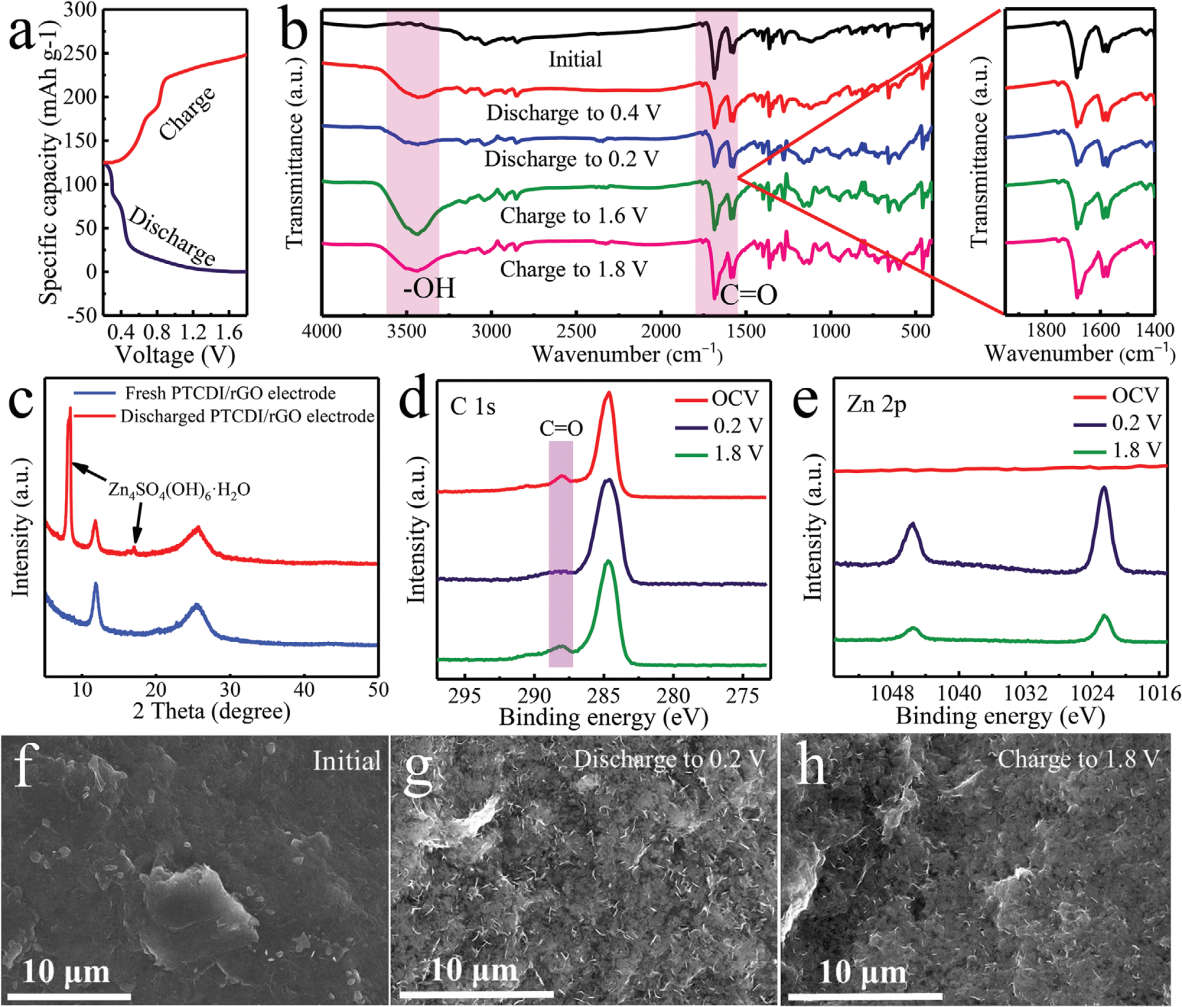
a) The charge/discharge curve of PTCDI/rGO batteries at a current density of 100 mA g^−1^. b) FTIR spectrum of PTCDI/rGO at different states. c) XRD patterns of the PTCDI/rGO electrode before and after cycles. d,e) XPS spectrum of PTCDI/rGO at different states of C 1s and Zn 2p. SEM images of PTCDI/rGO at f) initial state, g) discharge to 0.2 V, and h) charge to 1.8 V.

The interaction and displacement of the protons and Zn^2+^ with the PTCDI were in agreement with the result of the ex situ FTIR. Along with the proceeding discharging, the peak ascribed to hydroxyl showed a tendency from increase to decrease until vanishing away, coupled with weakened peak related to the carbonyl groups. Interestingly, with the proceeding charging, the change trend of the hydroxyl peak is the same as discharge process, accompanied with enhancing the peak of the carbonyl groups, which has been explained by previous reports.^[^ ^32^, ^33^ ^]^ The water participant phase transfer mechanism was also indirectly evidenced by XRD pattern (Figure 5c) and SEM images of PTCDI/rGO (Figure 5f–h). The typical phase and flake‐like morphology of Zn_4_SO_4_(OH)_6_·5H_2_O appeared at fully discharged states for PTCDI/rGO electrode, but it reversed to disappeared during full charge process, which came from the reaction of OH^−^ with ZnSO_4_ and H_2_O, while H^+^ in aqueous electrolyte diffused into PTCDI/rGO compounds to interact with PTCDI. This analysis was affirmed by energy‐dispersive X‐ray spectroscopy (EDS) spectrum (Figure S13, Supporting Information) and elemental mapping images (Figure S14, Supporting Information). As shown in Figures S15 and S16 (Supporting Information), the reversibility of this conversion reaction was also validated by the ex situ FTIR and SEM images of the fully discharged and charged stages at the different number of cycles. It was worth noting that the abovementioned conversion mechanism could be conducive to the fast charge transfer kinetics owing to the coparticipation of proton.

The electrochemical mechanism along the overall charge/discharge process was also testified by the corresponding ex situ XPS (Figure 4d,e and Figure S17 (Supporting Information)). Compared with the original compounds, the appearance of intense Zn signal revealed the successful chemical connection between Zn and PTCDI at the discharge state of 0.2 V. Nevertheless, after the end of charging, the intensity of Zn signal weakened to a certain extent, but not completely disappeared, indicating that Zn^2+^ were taken off from PTCDI with slight irreversibility. In addition, in the XPS spectra of C 1s, the peaks belonging to the C=O bond faded away, and subsequently increased during the whole cycling process, respectively. The test outcomes verified that the redox center was the carbonyl group, where the interaction of Zn^2+^ with PTCDI and the fracture of the C=O bonds came up simultaneously.

Inspired by superior rate capability and good cycling stability of PTCDI, we fabricated the rocking‐chair aqueous Zn‐ion full battery with the combination of the metal‐free PTCDI/rGO anode, 3 m ZnSO_4_ electrolyte, and pre‐embedded Zn Prussian blue cathode (**Figure** **6**a). The selected PB with a cubic structure has exhibited the high potential and low cost in the other aqueous Zn‐ion battery.^[^ ^10^, ^34^, ^35^ ^]^ As illustrated in Figure 6b, the PB cathode had a discharge capacity of 63 mAh g^−1^ and the discharge curves had three distinct plateaus. After Zn insertion, the crystal changed into hexagonal structure, and the particle size turned slightly larger than the precursor PB, symbolizing the well implantation of Zn and that the active material became the new construction (Figures S18–S20, Supporting Information). Figure 6c showed the charge/discharge profile of the Zn‐inserted PB//PTCDI/rGO full cell at the current density of 200 mA g^−1^. The full battery displayed a capacity of 193 mAh g^−1^ (based on the mass of active anode) with an average working voltage of 0.95 V, where the energy density achieved was 44 Wh kg_total_^−1^ (based on the total active mass of both electrodes). Additionally, we also explored the cycling performance of this aqueous full battery at 200 mA g^−1^ (Figure 6d). The discharge capacity reached 48 mAh g_total_^−1^, and gradually decayed to 36 mAh g_total_^−1^ with the capacity retention of 75% over 150 cycles. The remarkable cycling performance of the full battery could originate from high rate capability versus low solubility and reversible structural evolution of PTCDI/rGO. Oppositely, while Zn was used as the counter electrode, the assembled PB//Zn battery suffered from severe capacity degradation, and only remained at a capacity of 9 mAh g^−1^ after 150 cycles (Figure S21, Supporting Information). The morphologies of the cycled Zn anode demonstrated the uneven Zn stripping/plating process and the formation of incompact microflakes (Figure S22, Supporting Information), which was responsible for the capacity decay. To highlight the electrochemical advantages of the PTCDI/rGO, a comparison table was exhibited in Table S1 (Supporting Information). The substitution of organic anodes with Zn metal may lead to new direction for practical applications in aqueous Zn‐ion full battery.

**Figure 6:**
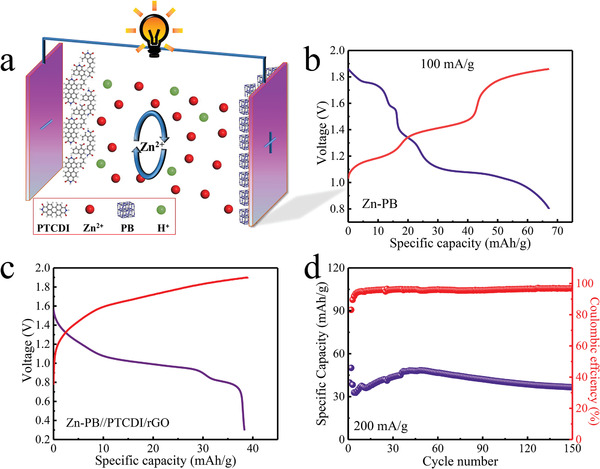
a) Schematic diagram of the full battery. b) The charge/discharge curve of PB//Zn battery at a current density of 100 mA g^−1^. c) The charge/discharge curve of the PB//PTCDI/rGO battery at a current density of 200 mA g^−1^. d) Cycling performance of the PB//PTCDI/rGO full battery at a current density of 200 mA g^−1^.

In conclusion, we develop the novel dendrite‐free organic anodes with the merits of cost‐effectiveness and durability for aqueous Zn‐ion batteries. The synthesized PTCDI/rGO electrode not only improves the stability of ZIBs by overcoming uneven Zn stripping/plating, but shows superior rate capability and a long cycling life span owing to a protons and zinc ions coparticipant phase transfer mechanism (130 mA h g^−1^ at 3000 mA g^−1^ with the capacity retention of 97% after 1500 cycles). Furthermore, the density functional theory was applied to explain the low potential of PTCDI. In addition, the full battery assembled with the PTCDI/rGO as anode and commercial PB as cathode displays a high capacity of 193 mAh g^−1^ and a suitable operation voltage of 0.95 V at 200 mA g^−1^ (based on anode), yet maintains 75% capacity at 200 mA g^−1^ with the cycling life span of 150 times. This work could provide new vision for organic electrode design and pave the way for excavating more organic compounds as anodes on Zn‐ion battery field.

## Conflict of Interest

The authors declare no conflict of interest.

## Supporting information

Supporting Information
