# Supplementary material for: Building High Rate Capability and Ultrastable Dendrite‐Free Organic Anode for Rechargeable Aqueous Zinc Batteries
Source: Adv Sci (Weinh). 2020 Jun 25;7(14):2000146. doi: 10.1002/advs.202000146 (PMC7375244; doi:10.1002/advs.202000146)
Supplement: Supplementary file 1 — Supporting Information [file ADVS-7-2000146-s001.pdf]

Copyright WILEY-VCH Verlag GmbH & Co. KGaA, 69469 Weinheim, Germany, 2020.

Supporting Information

Building High Rate Capability and Ultrastable Dendrite-free Organic Anode for Rechargeable Aqueous Zinc Batteries

Nannan Liu, Xian Wu, Yu Zhang, Yanyou Yin, Chengzhi Sun, Yachun Mao, Lishuang Fan and Naiqing Zhang**

Experimental Section

Material preparation.

Preparation of PTCDI/rGO: The rGO (reduced graphene oxides) was firstly synthesized in the manner of the ascorbic acid reduction. Namely, 70mL of GO (1 mg/mL) was mixed with 25 mg ascorbic acid, then kept stirring for 60 min at 95 ^{o}C to gain rGO suspension (The GO was typically prepared by modified Hummers method). Next, the PTCDI powders were uniformly dispersed in 10 mg/mL of sulfuric acid by ultrasonic treatment for 30 min. After that, the rGO was added to the above solution arising from the chemical oxidation polymerization at the interface between the PTCDI and rGO via π-π interaction. As continuing interaction diffusion, the self-assembly structure of the PTCDI anchored on rGO was entirely established. Subsequently, the obtained powders were followed by centrifuging and freeze drying to yield final products. The mass ratio of PTCDI and rGO in the composite was 7:2.

Material Characterization: The morphology and microstructure of the synthesized PTCDI/rGO powders were characterized by scanning electron microscope (SEM; Hitachi, SU 8010) and X-ray power diffractometer equipped with Cu Ka radiation (XRD, PANalytical X'Pert PRO, Cu Kα radiation) at room temperature. The surface chemical state and constituent of the electrode materials were examined using X-ray photoelectron spectroscopy

(XPS, ESCLAB 250Xi) and Raman spectrometry (LabRAM Xplora, excitement wavelength 532 nm). Fourier transform infrared spectroscopy (FT-IR) spectrum was carried out with a Nicolet is50 FT-IR Spectrometer using KBr pellets.

Electrochemical Measurement: The electrochemical studies were displayed via 2023 coin-type cells. The cells were fabricated by employing PTCDI/rGO as the cathode, the zinc foil as the anode, the glass fiber infiltrated with aqueous 3 M $ZnSO_4$ as separator, which was sandwiched between the electrode plates. The PTCDI/rGO electrode was prepared by mixing the active materials and PVDF (poly(vinylidenefluoride)) with a weight ratio of 90:10 in NMP (N-methylpyrrolidone) solvent, then pasted onto carbon papers. The areal loading density of the PTCDI/rGO cathode is up to 2-3 mg cm^{-2}. The galvanostatic charge-discharge (GCD) progress of the cells was measured on the Neware test system in the voltage range of 0.2-1.8 V versus Zn^{2+}/Zn. The electrochemical workstation was utilized to proceed with electrochemical impedance spectra and cyclic voltammetry tests. The cathode was assembled in the same way, the potential windows of pre-embedded Zn was in the range from 1-1.8 V versus Zn^{2+}/Zn, and the capacity was caculated based on the mass of PTCDI. The full batteries were assembled similarly as half batteries, but with Zn-PB as cathode and PTCDI/rGO as anode, the mass radio of the two electrodes was approximately 3:1.

The ion diffusion coefficients (*D*) were estimated through Galvanostatic Intermittent Titration Technique (GITT) and calculated according to the following equation:

$$D = \frac{4L^2}{\pi\tau}\left(\frac{\Delta E_S}{\Delta E_t}\right)^2$$

Where τ corresponds to the galvanostatic current pulse time (s); L is related to the thickness of the active material, approximately equals to the diffusion distance; ΔE_s and ΔE_t represent the change value of quasi-equilibrium potential (V) resulted from the current pulse and the voltage change (V) during the current pulse, respectively.

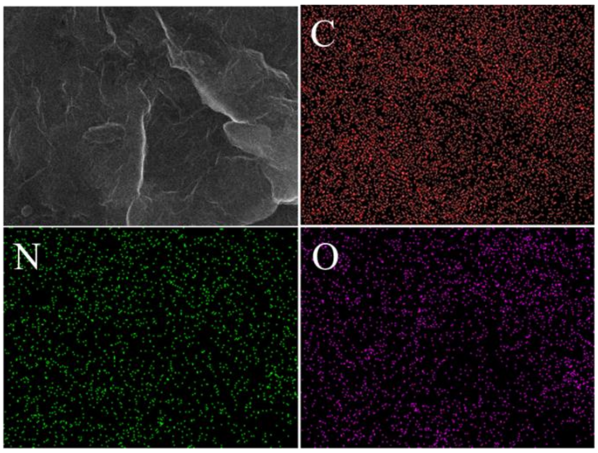

Figure S1. Mapping images of the PTCDI/rGO.

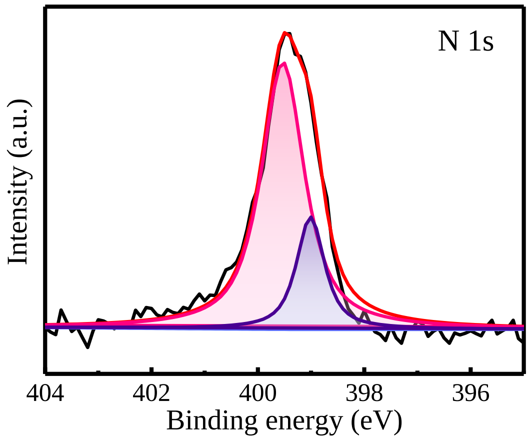

Figure S2. N 1s XPS spectra of the PTCDI/rGO.

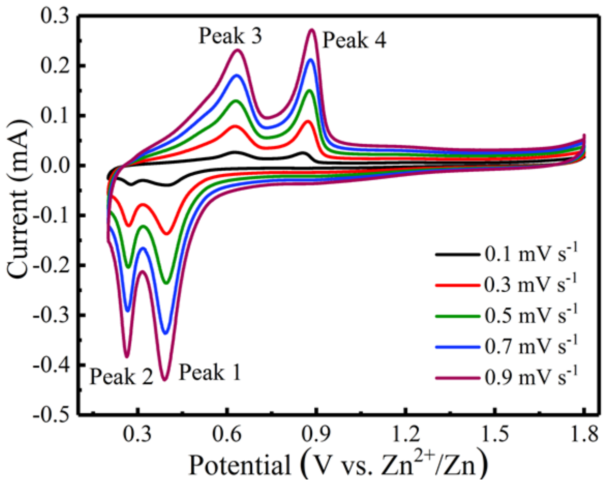

Figure S3. CV curves at different scan rates.

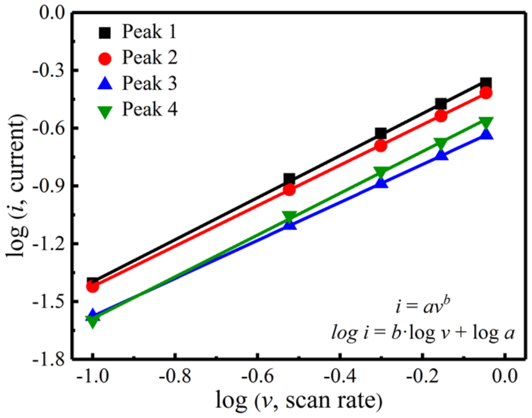

Figure S4. The logarithm dependence of peak current density and scan rate of the PTCDI/rGO.

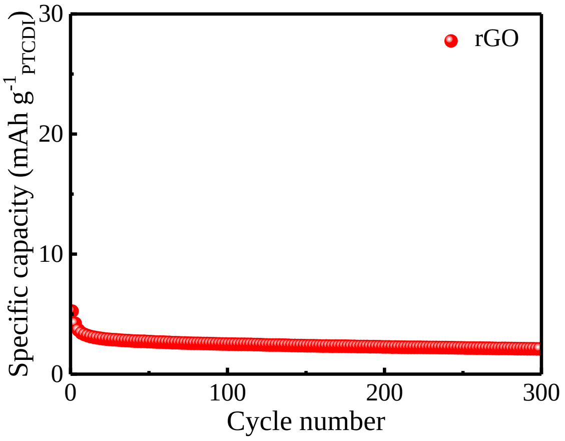

Figure S5. Cycling performance of the rGO electrodes at 100 mA g^{-1}.

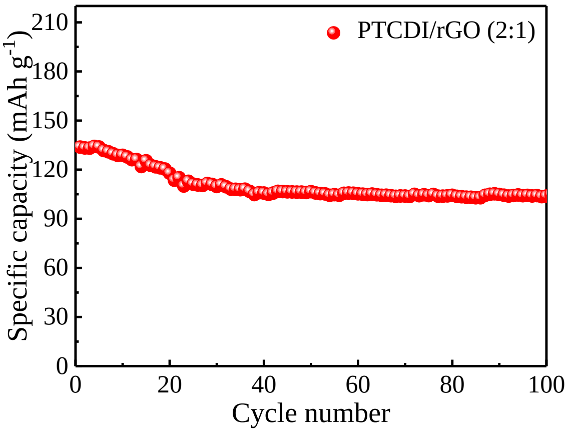

Figure S6. Cycling performance of PTCDI/rGO (2:1) electrodes at 100 mA g^{-1}.

Figure S6 showed The cyclic performances with different rGO content PTCDI/rGO composite (the mass ratio of PTCDI and rGO is 2:1). Although the PTCDI/rGO (2:1) showed

comparable performance to the PTCDI/rGO (7:2) based on the mass of PTCDI, and the capacity contribution of rGO substrates was negligible, the excessive addition of rGO would reduce loading mass of active material and cause energy density loss. Therefore, in the absence of additional conductive agent, 7:2 was considered to be the selective ratio.

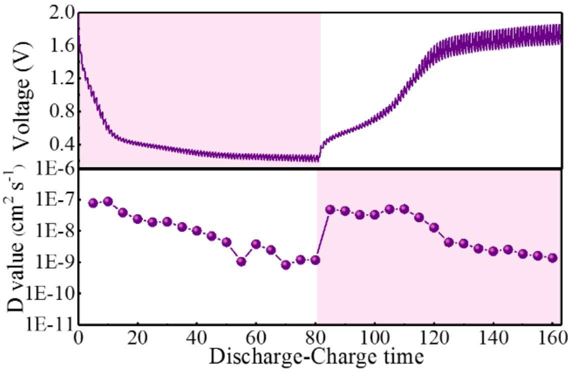

Figure S7. The GITT and diffusivity coefficient of PTCDI/rGO cathode at various states of discharge-charge during GITT measurements.

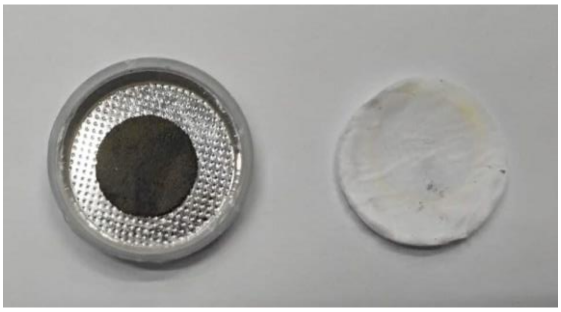

Figure S8. Optical image of the cycled PTCDI/rGO and separator.

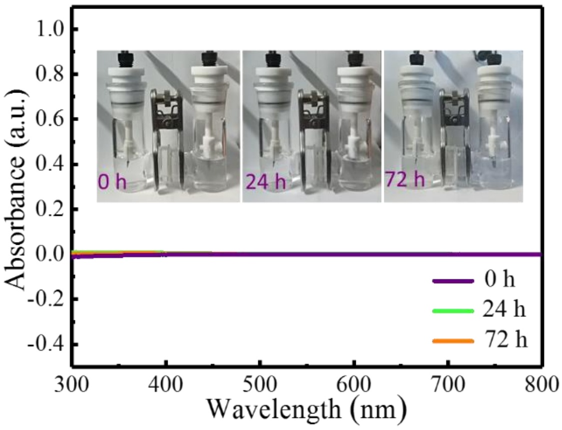

Figure S9. UV-vis spectrum of electrolyte at different electrochemical cycle time.

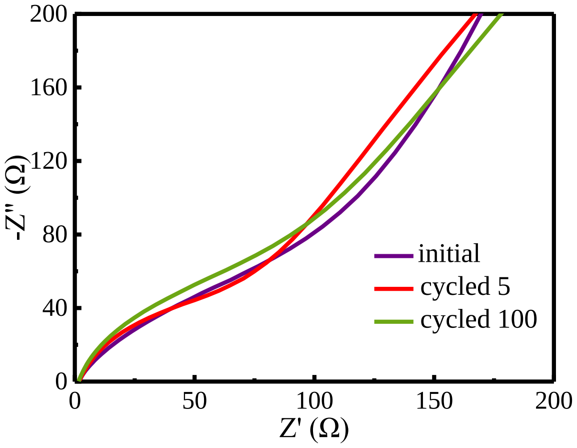

Figure S10. EIS of different electrochemical cycle time.

**TCBQ (Chloranil)**	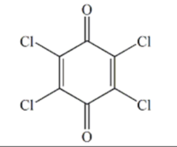	**PTCDA** (3,4,9,10-Perylenetetracarbo-xylic Dianhydride)	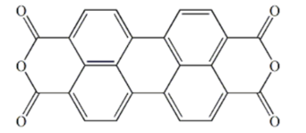
**PBQ (Benzoquinone)**	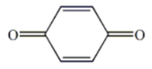	**PTCDI-O**	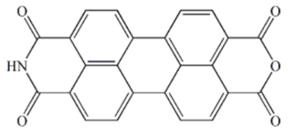
**AQ (Anthraquinone)**	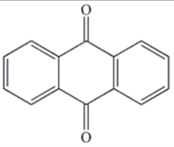	**PTCDI** (perylene-3,4,9,10-tetracarboxylic diimide)	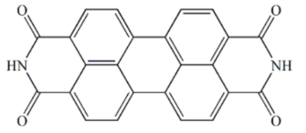

Figure S11. The chemical compositions of caculated organic molecules.

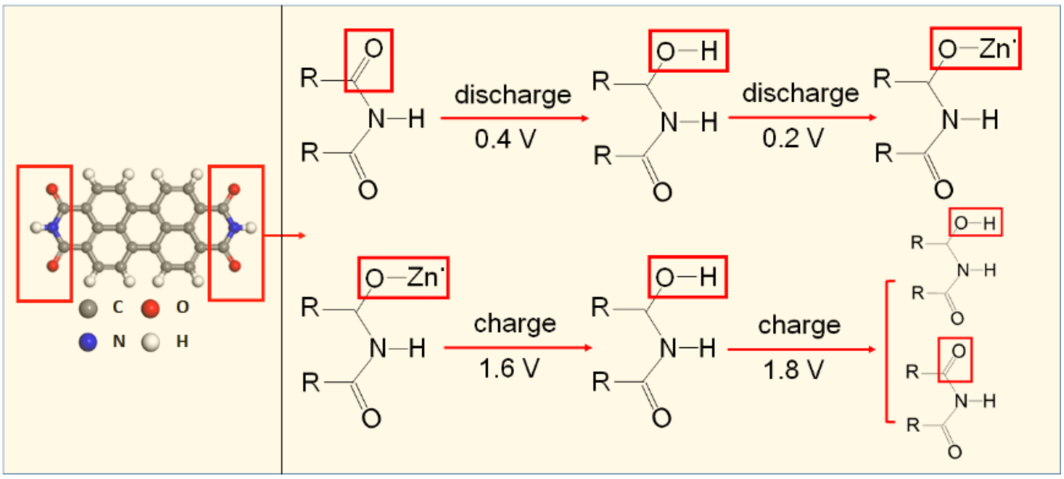

Figure S12. The electrochemical reaction mechanism at charge/discharge process.

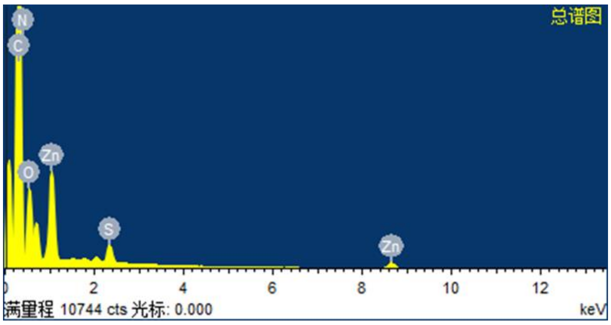

Figure S13. EDS of cycled PTCDI/rGO.

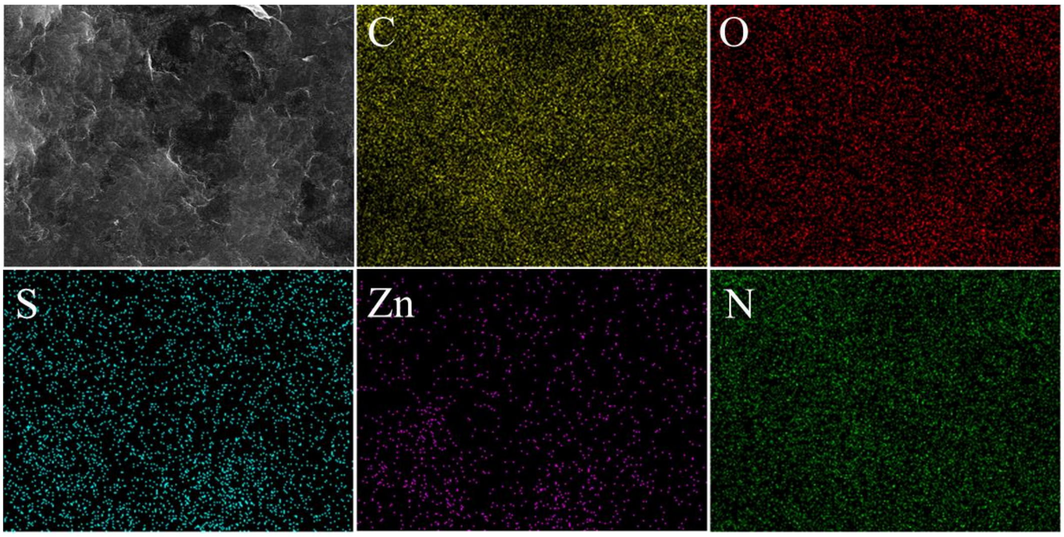

Figure S14. SEM and mapping images of cycled PTCDI/rGO.

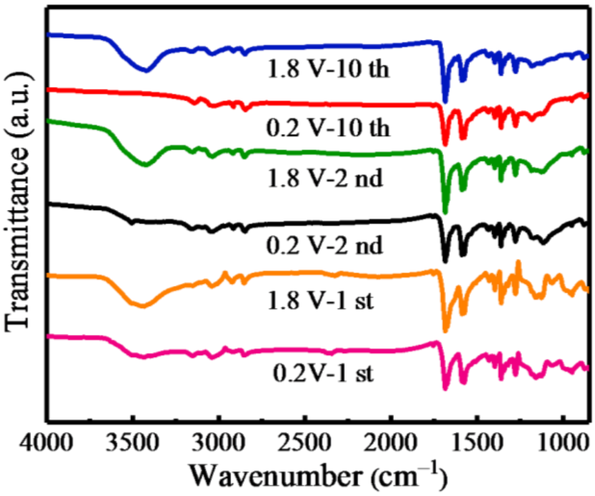

Figure S15. Ex-situ FTIR of PTCDI/rGO at different electrochemical cycle states.

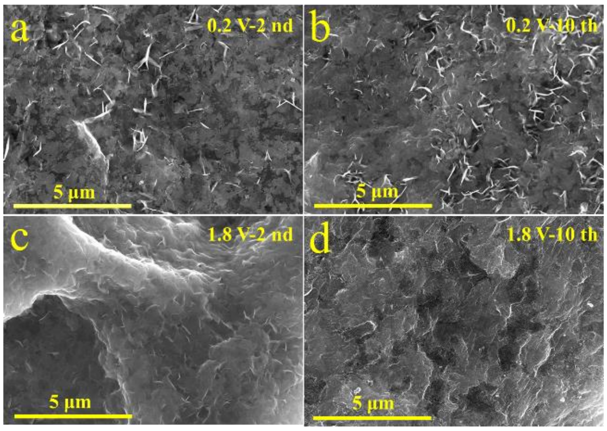

Figure S16. SEM images of the PTCDI/rGO at different electrochemical cycle states, a) discharge to 0.2 V at the second cycle, b) discharge to 0.2 V at the 10th cycle and c) charge to 1.8 V at the second cycle, d) charge to 1.8 V at the 10th cycle.

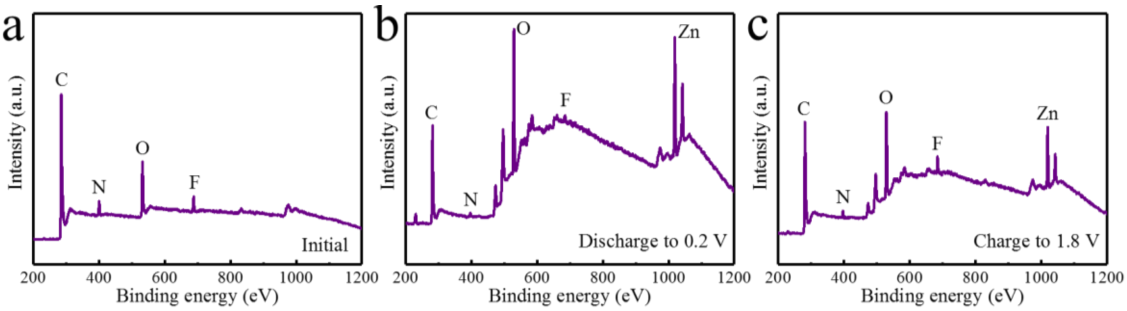

Figure S17. XPS full survey spectrum of PTCDI/rGO at a) initial state, b) discharge to 0.2 V, and c) charge to 1.8 V.

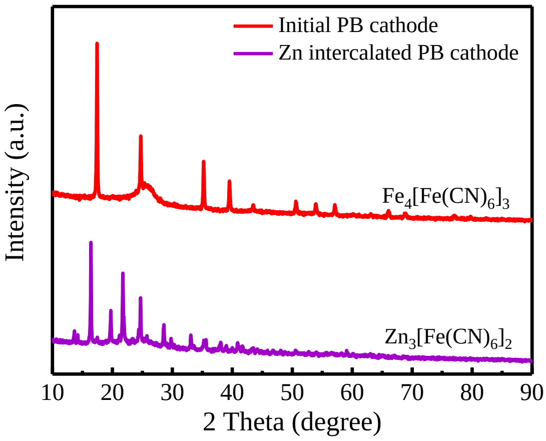

Figure S18. XRD patterns of PB cathode and Zn-inserted PB cathode.

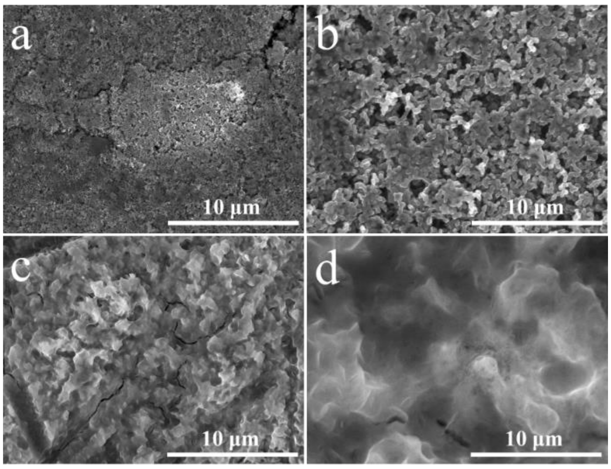

Figure S19. SEM images of (a, b) PB cathode and (c, d) Zn-inserted PB cathode.

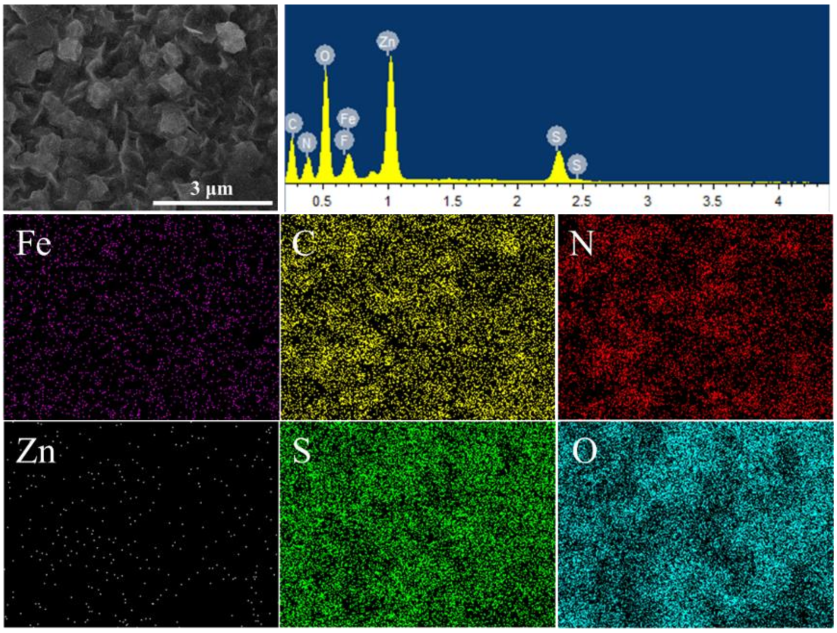

Figure S20. EDS and mapping images of cycled PB cathode.

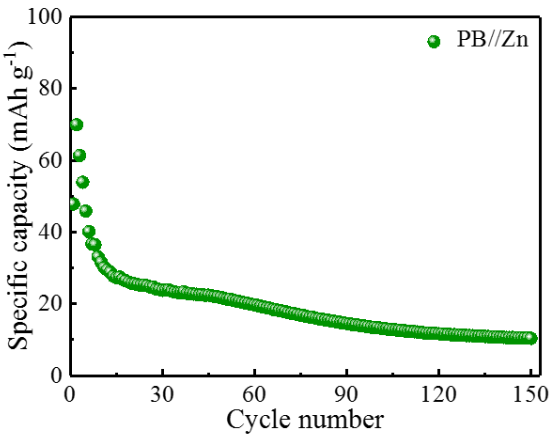

Figure S21. Cycling performance of PB electrodes at 200 mA g^{-1}.

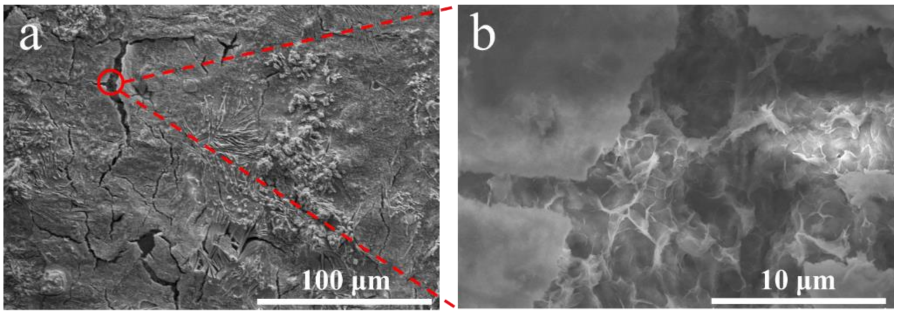

Figure S22. Different magnification SEM images of Cycled Zn electrodes when PB as cathode.

Table S1. Electrochemical performance based on different electrodes.

	Position	Voltage	Rate performance	Capacity retain	Reference
$Na_{0.14}TiS_2$	Anode	0.3 V	58 mAh g^{-1} (2 A g^{-1})	77 % (0.5 A g^{-1})	S[1]
Mo_6S_8	Anode	0.34 V	54 mAh g^{-1} (0.52 A g^{-1})	90 % (0.6 A g^{-1})	S[2]
C4Q	Cathode	1 V	172 mAh g^{-1} (1 A g^{-1})	87 % (0.5 A g^{-1})	S[3]
PBQS	Cathode	0.95 V	126 mAh g^{-1} (1.94 A g^{-1})	53 % (0.1 A g^{-1})	S[4]
MOCP-H	Cathode	1.2 V	35.6 mAh g^{-1} (1 A g^{-1})	93 % (0.6 A g^{-1})	S[5]
p-chloranil	Cathode	1.1 V	118 mAh g^{-1} (0.22 A g^{-1})	70 % (0.2 A g^{-1})	S[6]
PTCDI/rGO	Anode	0.38 V	121 mAh g^{-1} (5 A g^{-1})	95 % (0.5 A g^{-1})	This work

Reference

[1] W. Li, K. Wang, S. Cheng, K. Jiang, *Adv. Energy Mater.* **2019**, 9, 1900993.

[2] Y. Cheng, L. Luo, L. Zhong, J. Chen, B. Li, W. Wang, S. X. Mao, C. Wang, V. L. Sprenkle, G. Li, J. Liu, *ACS Appl. Mater. Interfaces* **2016**, 8, 13673.

[3] Q. Zhao, W. Huang, Z. Luo, L. Liu, Y. Lu, Y. Li, L. Li, J. Hu, H. Ma, J. Chen, *Sci. Adv.* **2018**, 4, 1761.

[4] G. Dawut, Y. Lu, L. Miao, J. Chen, *Inorg. Chem. Front.* **2018**, 5, 1391.

[5] S. Wu, Y.-F. Wang, W.-L. Liu, M.-M. Ren, F.-G. Kong, S.-J. Wang, X.-Q. Wang, H. Zhao, J.-M. Bao, *Inorg. Chem. Front.* **2018**, 5, 3067.

[6] D. Kundu, P. Oberholzer, C. Glaros, A. Bouzid, E. Tervoort, A. Pasquarello, M. Niederberger, *Chem. Mater.* **2018**, 30, 3874.
